# Dietary linoleic acid intake in relation to breast cancer: A case-control study

**DOI:** 10.34172/hpp.2023.27

**Published:** 2023-09-11

**Authors:** Muhammad Reza Joya, Sina Naghshi, Omid Sadeghi, Sanaz Benisi-Kohansal, Leila Azadbakht, Keyhan Lotfi, Alireza Ostadrahimi, Helda Tutunchi, Ahmad Esmaillzadeh

**Affiliations:** ^1^Department of Community Nutrition, School of Nutritional Sciences and Dietetics, Tehran University of Medical Sciences, Tehran, Iran; ^2^Nutrition Department, Kabul University of Medical Sciences, Kabul, Afghanistan; ^3^Student Research Committee, Tabriz University of Medical Sciences, Tabriz, Iran; ^4^Nutrition Research Center, Department of Clinical Nutrition, School of Nutrition and Food Science, Tabriz University of Medical Sciences, Tabriz, Iran; ^5^Endocrine Research Center, Tabriz University of Medical Sciences, Tabriz, Iran; ^6^Obesity and Eating Habits Research Center, Endocrinology and Metabolism Molecular -Cellular Sciences Institute, Tehran University of Medical Sciences, Tehran, Iran; ^7^Department of Community Nutrition, School of Nutrition and Food Science, Isfahan University of Medical Sciences, Isfahan, Iran

**Keywords:** Breast neoplasms, Linoleic acid, Diet, Case-control studies, Fatty acids

## Abstract

**Background:**

The present study aimed to investigate the association between dietary linoleic acid (LA) intake and breast cancer in women.

**Methods:**

In this population-based case-control study, we enrolled 350 pathologically confirmed breast cancer cases and 700 controls which were matched with cases in terms of age and socioeconomic status. Dietary intakes were assessed using a 106-item Willett-format semi-quantitative dish-based food frequency questionnaire (DS-FFQ). Odds ratios (ORs) and the corresponding 95% confidence intervals (CIs) were estimated.

**Results:**

A significant inverse association was found between LA intake and odds of breast cancer (OR: 0.41, 95% CI: 0.30-0.56). After adjusting for potential confounders, women in the highest tertile of dietary LA intake were 48% less likely to have breast cancer compared with those in the lowest tertile (OR: 0.52, 95% CI: 0.28-0.95). Such a significant inverse association was also seen among normal-weight women (OR: 0.29, 95% CI: 0.14-0.63), and premenopausal women (OR: 0.15, 95% CI: 0.02-0.95).

**Conclusion:**

The findings of current study provide evidence for a protective role of LA against breast cancer particularly among normal-weight and premenopausal women. Prospective studies are needed to confirm this association.

## Introduction

 Breast cancer is one of the most common cancers, impacting 2.1 million women each year, it also causes a huge number of cancer-related deaths among women, accounting for 15% of cancer deaths in 2018.^1^ Breast cancer imposes a substantial economic burden on patients and healthcare systems.

 For the past few decades, evidence from epidemiological studies has suggested that diet, lifestyle and inflammation undisputedly play a major role in the etiology and progression of breast cancer. ^2^ Dietary fat is one of the most important dietary factors closely linked to the risk of breast cancer. ^3^ Among subtypes of dietary fat, linoleic acid (LA), as a major omega-6 fatty acid in the diet, has received particular attention.^4^ Previous studies have examined the association of n-3 polyunsaturated fatty acids (PUFAs) with cancer risk, but the association of n-6 PUFAs intake with cancer risk has been studied less extensively. LA is associated with the risk of inflammation, which is recognized as a strong risk factor for many cancers.^5^ A recent summary of prospective cohort studies reported a null association between dietary LA intake and risk of breast cancer. ^4^ However, another meta-analysis of case-control studies concluded that high dietary LA intake was marginally associated with a reduced risk of breast cancer.^6^ Therefore, it seems that evidence linking dietary LA intake to risk of breast cancer remains conflicting.

 The dietary intake of people in the Middle East can provide a unique opportunity to examine the link between diet and disease. In this area, dietary fat is consumed at the level of dietary reference intake (DRI); however, the type of dietary fat has always been a concern in this region. In addition, nutrition transition in Middle-Eastern countries is associated with a shift from animal fat intake to vegetable oil consumption. Therefore, dietary consumption of LA has increased in these countries in recent years. Such dietary characteristics make it reasonable to examine the contribution of dietary fat intake to human health. Although the role of other macronutrients in different cancers has been examined before,^7,8^ no study is available linking breast cancer and dietary LA in these countries. Therefore, the current study was designed to investigate whether LA was associated with breast cancer risk among Iranian women.

## Materials and Methods

###  Study population

 In apopulation-based case-control study, we recruited female participants aged more than 30 years in Isfahan, Iran. All participants were residents of Isfahan, Iran. All cases were individuals diagnosed with breast cancer during the last six months by physical examination and mammography findings. Breast cancer cases were recruited from July 2013 to July 2015 from those that were referred to private clinics or hospitals in Isfahan, Iran. The sample size calculation was based on the type I error of 5% and the study power of 80%. Considering the common ratio of 0.25 and the ratio of controls to cases as 2, we reached almost 350 patients with breast cancer and 700 apparently healthy controls. Breast cancer patients were defined as those who had the primary incident breast tumor with invasive behavior and its histology was available from medical records. Individuals with a history of any type of neoplastic lesion or cysts (except current breast cancer), those who had undergone hormone replacement therapy, and those following a specific diet were excluded from participation in the study. Controls were selected from healthy women who had no relationship with breast cancer cases and had no family history of breast cancer. Cases and controls were matched in terms of age and socioeconomic status. The inclusion criteria for controls included being female, of Iranian nationality, having no special diet or hormone replacement therapy, and no prior history of malignancy, cysts, or medical disorders. Controls were randomly selected from apparently healthy women by multistage cluster random sampling method. Individuals who were not relatives of patients with breast cancer who attended Primary Health Care Centers for their annual personal checkup or attended to receive required information about their children (i.e., growth monitoring, vaccination, …) were selected. Two healthcare centers were randomly selected from several centers in Isfahan, taking into account the covered population and considering the attendance of women in these centers, the required sample was recruited. Finally, 350 cases and 700 controls were recruited for the study. Written informed consent was obtained from all participants. The study was ethically approved by the Ethical Committee of Tehran University of Medical Sciences, Tehran, Iran (Ethics code: IR.TUMS.VCR.REC.1397.1036).

###  Assessment of dietary intakes

 Dietary intake was examined using a 106-item Willett-format semi-quantitative dish-based food frequency questionnaire (DS-FFQ) which was designed and validated specifically for Iranian adults.^9^ In face-to-face interviews, participants were asked to report their average frequency intakes of foods and dishes over the past year on a daily, weekly, or monthly basis. The DS-FFQ contained five categories of foods and dishes: (1) mixed dishes (cooked or canned, 29 items); (2) fruits and vegetables (22 items); (3) carbohydrate-based foods (different types of bread, biscuits, cakes, and potatoes, 10 items); (4) dairy products (dairies, butter, and cream, 9 items); and (5) miscellaneous food items and beverages (including sweets, nuts, fast foods, desserts and beverages, 36 items). Participants were asked about their dietary intake of foods and mixed dishes based on nine multiple-choice frequency response categories ranging from “never or less than once a month” to “12 or more times per day.” The frequency response categories for the food list varied from 6 to 9 choices. We omitted the high-frequency categories for foods consumed infrequently, while for common foods with high consumption, the number of multiple-choice categories increased. Finally, we converted the daily intake of all food items to grams per day using household measures.^10^ We analyzed the nutrient intake for each person using Nutritionist IV software, which was modified for Iranian foods. Our previous study indicated that this FFQ provided valid and reliable measures of long-term dietary intake.^9^

###  Assessment of breast cancer

 All cases were women with newly diagnosed stage I-IV breast cancer of Iranian nationality, for whom the in-situ or invasive status of breast cancer was confirmed by physical examination and mammography.

###  Assessment of other variables

 Information on lifestyle and other potential risk factors for breast cancer was assessed through an interview-based questionnaire, including age, alcohol intake (yes/no), smoking (non-smoker/smoker), marital status (single/married), menopause status (premenopausal/postmenopausal), family history of breast cancer (yes/no), disease history (yes/no), region (urban/rural) and education (educated/non-educated). In terms of anthropometric measurements, weight was measured to the nearest 100 g using digital scales with minimal clothes, and height was measured to the nearest 0.5 cm by a tape meter mounted on the wall while the subject was standing in a normal position without shoes. The body mass index (BMI) was calculated by dividing weight (kg) by height squared (m^2^). Physical activity was assessed using the short form of International Physical Activity Questionnaire (IPAQ), a simple questionnaire reflecting an individual’s current physical activity. In the current analysis, we categorized participants as having < 1 h/wk (physically inactive) or ≥ 1 h/wk (physically active) of physical activity.

###  Statistical analysis

 First, energy-adjusted intakes of dietary LA were calculated based on residual method. We used the independent samples *t*test and chi-square test to examine differences in terms of general characteristics between cases and controls.Then,we categorized participants based on tertile cut-off points of dietary LA intake. One-way analysis of variance (ANOVA) was used to determine differences in continuous variables across tertiles of dietary LA. The chi-square test was applied to examine the distribution of participants in terms of categorical variables across tertiles of dietary LA. We applied binary logistic regression to examine the association between dietary LA and breast cancer. In the first model, we adjusted for age and energy intake. In the second model, further adjustments were made for region (urban/rural), family history of breast cancer (yes/no), alcohol consumption (yes/no), smoking (non-smoker/smoker), physical activity (active/inactive) marital status (single/married), menopausal status (premenopausal/ postmenopausal), score of socioeconomic status (continuous) and disease history (yes/no). In the third model, we also controlled for monounsaturated fatty acids (MUFA), saturated fatty acids (SFAs), trans-fatty acids (TFAs), and vitamin E. In the final model, we also controlled for BMI to obtain an obesity-independent association between LA and breast cancer. In all analyses, individuals in the first tertile of dietary LA were considered the reference group. To obtain the overall trend of odds ratios (ORs) across increasing tertiles of dietary LA, we considered these categories as ordinal variables. The same analyses were also done stratified by menopausal status (premenopausal/ postmenopausal) and BMI categories (normal weight/overweight or obese). All statistical analyses were conducted using IBM SPSS Statistics software (IBM SPSS Statistics, Armonk, USA, version 23). *P* value < 0.05 was considered as a significant probability.

## Results

 General characteristics of cases and controls are shown in Table 1. This table has been published previously.^11^ Compared to controls, cases were older, had lower BMI, and were more likely to be current smoker and postmenopausal, and were less likely to be married and educated. No other significant difference was found between cases and controls.

**Table 1:** General characteristics of cases and controls

**Variables**	**Cases** **(n=350)**	**Controls** **(n=700)**	***P*** ** value** ^*^
Age (year)	65.2 ± 11.2	61.0 ± 10.3	< 0.001
Social economic score	12.8 ± 5.2	13.4 ± 5.5	0.09
BMI (kg/m^2^)	21.8 ± 4.8	25.5 ± 5.0	< 0.001
Region (urban) (%)	126 (36.0)	253 (36.1)	0.96
Marital status (married) (%)	261 (74.6)	618 (88.3)	< 0.001
Education (educated) (%)	61 (17.4)	202 (28.9)	< 0.001
Disease history (yes) (%)	36 (10.3)	61 (8.7)	0.40
Physical activity (inactive) (%)	111 (31.7)	241 (34.4)	0.38
Menopausal status (postmenopausal) (%)	309 (88.3)	542 (77.4)	< 0.001
Smoking (smoker) (%)	61 (17.4)	91 (13.0)	0.05
Alcohol consumption (yes) (%)	16 (4.6)	52 (7.4)	0.07

Data are presented as Means ± SD or n (%). Abbreviations: BMI: body mass index; SD: standard deviation. ^*^Obtained from independent sample t-test or chi-square, where appropriate.

 General characteristics of study participants across tertiles of LA are presented in Table 2.Participants in the top tertile of LA were younger, had greater BMI and socioeconomic score, were more likely to be alcoholics, educated, urban residents, and were less likely to be single and have a history of breast cancer than those in the bottom tertile. No other significant differences were observed in terms of other variables across categories of LA.

**Table 2:** General characteristics of the study participants across tertiles of dietary linoleic acid

	**Tertile1** **(n=350)**	**Tertile2** **(n=350)**	**Tertile3** **(n=350)**	***P*** ** value** ^*^
Age (year)	63.92 ± 10.56	61.96 ± 10.88	61.49 ± 10.95	0.007
BMI (kg/m^2^)	23.57 ± 5.39	24.44 ± 5.26	24.96 ± 5.13	0.002
Socioeconomic score	12.38 ± 5.04	12.90 ± 5.25	14.39 ± 5.87	< 0.001
Region (urban)	117 (33.4)	115 (32.9)	147 (42.0)	0.01
Marital Status (married)	284 (81.1)	292 (83.4)	303 (86.6)	0.26
Education (educated)	73 (20.9)	75 (21.4)	115 (32.9)	< 0.001
Disease history (yes)	40 (11.4)	33 (9.4)	24 (6.9)	0.11
Physical activity (inactive)	124 (35.4)	120 (34.3)	108 (30.9)	0.41
Menopausal status (post)	294 (84.0)	282 (80.6)	275 (78.6)	0.18
Smoking (smoker)	64 (18.3)	45 (12.9)	43 (12.3)	0.04
Alcohol consumption (yes)	11 (3.1)	18 (5.1)	39 (11.1)	< 0.001

Data are presented as Means ± SD or n (%). Abbreviations: BMI: body mass index; SD: standard deviation. ^*^Obtained from one-way ANOVA or chi-square, where appropriate.

 Dietary intakes of participants across tertiles of LA are indicated in Table 3. Individuals in the top tertiles of dietary LA had higher intakes of red meat, total protein, vegetables, total fiber, vitamin C, and lower intakes of carbohydrate, sucrose, trans fatty acids compared with those in the bottom tertile.

**Table 3:** Dietary intakes of study participants across tertiles of dietary linoleic acid

	**Tertile 1 (n=350)**	**Tertile 2 (n=350)**	**Tertile 3 (n=350)**	***P*** ** value** ^*^
Energy (kcal/d)	2040.41 ± 39.46	2260.30 ± 32.65	2554.24 ± 33.33	< 0.001
Carbohydrate (g/d)	322.08 ± 3.24	321.16 ± 2.33	308.52 ± 2.53	< 0.001
Protein (g/d)	69.43 ± 0.79	76.70 ± 0.81	86.35 ± 1.29	< 0.001
Total fat (g/d)	86.73 ± 1.40	83.12 ± 0.97	83.94 ± 0.96	0.06
Trans fatty acids (g/d)	0.51 ± 0.17	0.42 ± 0.10	0.31 ± 0.12	< 0.001
Whole grain (g/d)	322.17 ± 6.35	322.82 ± 7.46	305.64 ± 6.96	0.14
Total dairy (g/d)	240.67 ± 8.36	225.60 ± 7.62	228.22 ± 7.08	0.33
Fruits (g/d)	175.45 ± 7.37	156.62 ± 7.18	162.31 ± 7.76	0.18
Red meat (g/d)	12.09 ± 0.70	14.02 ± 0.99	15.30 ± 0.93	0.03
Vegetables (g/d)	66.47 ± 2.51	79.90 ± 3.48	96.47 ± 4.68	< 0.001
Fiber (g/d)	20.94 ± 0.27	22.50 ± 0.23	23.75 ± 0.26	< 0.001
Sucrose (g/d)	44.47 ± 3.21	38.82 ± 2.04	29.91 ± 1.66	< 0.001
Vitamin C (mg/d)	58.75 ± 1.51	60.29 ± 2.04	66.34 ± 2.20	0.01
Magnesium (mg/d)	445.65 ± 4.36	452.92 ± 4.17	456.81 ± 4.36	0.17

Data are presented as Means ± SE. Abbreviation: SE: standard error. ^*^Obtained from one-way ANOVA.

 Multivariable-adjusted ORs and 95% confidence intervals (CIs) for breast cancer across tertiles of energy-adjusted LA are shown in Table 4. A significant inverse association was found between dietary LA and odds of breast cancer (OR: 0.41; 95% CI: 0.30-0.56). This association remained significant after taking age, and energy intake into account. A significant inverse association was reached when we controlled for sociodemographic variables. Such a significant association was also seen when further adjustments were made for dietary SFA, TFA, MUFA, and vitamin E. This association did not change in the fully adjusted model in which we additionally adjusted for BMI, such that women in the highest tertile of LA had 48% lower odds of breast cancer compared with those in the lowest tertile (OR: 0.52; 95% CI: 0.28-0.95).

**Table 4:** Multivariable-adjusted odds ratios and 95% CIs for breast cancer across tertiles of dietary linoleic acid (n = 1050)

**Variables**	**Tertiles of dietary linoleic acid**			***P*** **-trend** ^*^
	**Tertile1**	**Tertile2**	**Tertile3**	
Crude	1	0.39 (0.28-0.54)	0.41 (0.30-0.56)	< 0.001
Model 1	1	0.31 (0.22-0.43)	0.24 (0.17-0.35)	< 0.001
Model 2	1	0.29 (0.20-0.42)	0.23 (0.15-0.33)	< 0.001
Model 3	1	0.37 (0.25-0.55)	0.47 (0.27-0.83)	0.001
Model 4	1	0.37 (0.24-0.57)	0.52 (0.28-0.95)	0.007

Data are presented as OR and 95% CI. Model 1: Adjusted for age and energy intake. Model 2: Additional adjustment for region, marital status, disease history, physical activity, family history of breast cancer, menopausal status, smoking, alcohol consumption, and socioeconomic status. Model 3: Additional adjustment for intakes of monounsaturated fatty acids, saturated fatty acids, trans-fatty acids, and vitamin E. Model 4: Additional adjustment for BMI. Abbreviations: BMI: body mass index; OR: odds ratio; CI: confidence interval. ^*^Obtained from binary logistic regression.

 When we performed stratified analysis based on menopausal status, a significant inverse association was found between dietary LA and odds of breast cancer in both postmenopausal (OR: 0.43; 95% CI: 0.30-0.60) and premenopausal women (OR: 0.39; 95% CI: 0.17-0.93) (Table 5). After taking age, BMI, intakes of energy, SFA, TFA, MUFA, vitamin E, and sociodemographic variables into account, this association remained significant among premenopausal women (OR: 0.15; 95% CI: 0.02-0.95) and became non-significant among postmenopausal women (OR: 0.77; 95% CI: 0.39-1.53).

**Table 5:** Multivariable-adjusted odds ratios for breast cancer across tertiles of dietary linoleic acid stratified based on menopausal and BMI status

**Variables**	**Tertiles of dietary linoleic acid**			***P*** **-trend** ^*^
	**Tertile1**	**Tertile2**	**Tertile3**	
**Menopausal status**				
Premenopausal				
n	56	68	75	
Crude	1	0.54 (0.24-1.24)	0.39 (0.17-0.93)	0.03
Model 1	1	0.38 (0.16-0.95)	0.19 (0.07-0.51)	0.001
Model 2^a^	1	0.40 (0.16-1.04)	0.18 (0.06-0.52)	0.002
Model 3	1	0.28 (0.10-0.79)	0.08 (0.02-0.39)	0.001
Model 4	1	0.20 (0.06-0.77)	0.15 (0.02-0.95)	0.03
Postmenopausal				
n	294	282	275	
Crude	1	0.38 (0.27-0.53)	0.43 (0.30-0.60)	< 0.001
Model 1	1	0.29 (0.20-0.42)	0.25 (0.17-0.37)	< 0.001
Model 2^a^	1	0.27 (0.18-0.40)	0.23 (0.15-0.35)	< 0.001
Model 3	1	0.38 (0.24-0.60)	0.67 (0.35-1.29)	0.07
Model 4	1	0.42 (0.26-0.68)	0.77 (0.39-1.53)	0.19
**BMI status**				
Normal weight				
n	234	206	183	
Crude	1	0.48 (0.33-0.70)	0.40 (0.27-0.60)	< 0.001
Model 1	1	0.33 (0.22-0.51)	0.21 (0.13-0.33)	< 0.001
Model 2^b^	1	0.31 (0.20-0.48)	0.18 (0.11-0.30)	< 0.001
Model 3	1	0.37 (0.23-0.62)	0.29 (0.14-0.63)	< 0.001
Overweight or obese				
n	116	144	167	
Crude	1	0.24 (0.12-0.48)	0.57 (0.33-0.99)	0.07
Model 1	1	0.22 (0.10-0.45)	0.37 (0.20-0.69)	0.003
Model 2^b^	1	0.18 (0.08-0.39)	0.37 (0.19-0.72)	0.006
Model 3	1	0.30 (0.12-0.74)	2.02 (0.69-5.93)	0.29

Data are presented as OR and 95% CI. Model 1: Adjusted for age and energy. ^a^ Model 2: Additional adjustment for region, marital status, disease history, physical activity, family history of breast cancer, smoking, alcohol consumption, and socioeconomic status. ^b^ Model 2: Additional adjustment for region, marital status, disease history, physical activity, family history of breast cancer, smoking, alcohol consumption, socioeconomic status and menopausal status. Model 3: Additional adjustment for intakes of monounsaturated fatty acids, saturated fatty acids, trans-fatty acids, and vitamin E. Model 4: Additional adjustment for BMI. Abbreviations: BMI: body mass index; OR: odds ratio; CI: confidence interval. ^*^Obtained from binary logistic regression

 In stratified analyses based on BMI status, LA was inversely associated with odds of breast cancer in normal-weight women before (OR: 0.40; 95% CI: 0.27-0.60) and after (OR: 0.29; 95% CI: 0.13-0.63) considering potential confounders. Such a significant inverse association was also seen among women with overweight or obesity in the crude model (OR: 0.57; 95% CI: 0.33-0.99). However, this association became non-significant after adjusting for potential confounders (Table 5).

## Discussion

 In the current study, we found that a greater intake of dietary LA was associated with reduced odds of breast cancer. This association remained significant even after taking potential confounders into account. Such a significant inverse association was also seen in normal-weight and premenopausal women. To the best of our knowledge, this study is the first in the Middle East to examine the association between LA intake and odds of breast cancer.

 Quality of dietary fat intake is much more important than its quantity to predict the risk of chronic conditions, including breast cancer.^12^ Unlike western communities, it seems that the quantity of dietary fat intake in Middle-Eastern countries are at the level of DRI; however, there are great concerns about the quality of dietary fat in this region. For instance, in some countries in this area, it has been shown that people are taking more than 4% of their energy from trans fats, which is much higher than the recommended levels.^13^ Another concern is changing patterns of dietary fat intake in these countries. In parallel to the westernization of lifestyle in this area, which might be associated with a greater intake of dietary fats, increased awareness about the difference between hydrogenated and non-hydrogenated fats has resulted in reduced consumption of hydrogenated vegetable oils and rather increased intake of non-hydrogenated vegetable oils, which might be associated with increased consumption of dietary LA.^14^ This fatty acid has earlier been shown to contribute to reduced risk of several chronic conditions, including diabetes^15^ and even mortality.^16^ We found an inverse association between dietary LA and odds of breast cancer. This finding was in line with an Italian case-control study on a large sample of women in which LA intake was strongly associated with a reduced risk of breast cancer.^17^ Such a significant inverse association was also seen in a case-control study in Uruguay.^18^ Contrary to our findings, two cohort studies revealed no significant association between dietary LA and risk of breast cancer.^5,19^ Different LA food sources, variations in LA intake, and various dietary assessment techniques across studies may all contribute to discrepant findings regarding the association between LA and breast cancer. Another potential explanation for the discrepancy might be related to the lack of consideration of covariates across different studies.

 In the current study, we failed to find any significant association between dietary LA and breast cancer among postmenopausal women. However, a significant inverse association was reached among premenopausal women. In a case-control study from US, researchers reported a significant inverse association between LA intake and premenopausal breast cancer risk.^20^ Similar non-significant results were seen for postmenopausal women in other cohort studies.^21,22^ In contrast, in the Nurses’ Health Study, no significant association was reported between LA and breast cancer among either premenopausal or postmenopausal women.^23^

 Unlike normal-weight women, we found no significant association between dietary LA and breast cancer in overweight or obese participants. This might be partly due to hormonal systems, including the insulin and insulin-like growth factor (IGF) axis, sex steroids, and adipokines. For postmenopausal breast cancer, this might be explained by the high circulating estrogen levels in obese women which are produced by aromatase in adipose tissue.^24^ In premenopausal obese women, circulating levels of total IGF-I were associated with a higher risk of breast cancer.^25^ Moreover, long-standing hyperinsulinemia, which is seen in obesity, promotes tumorigenesis in estrogen-sensitive tissues.^26^ Adiponectin is an adipokine that is inversely correlated with BMI. Some studies reported an inverse association between adiponectin levels and breast cancer risk.^27^

 Our previous report from the same population indicates that nuts and legumes are negatively associated with odds of breast cancer.^28^ Also, we have previously found an inverse association between low-fat dairy and breast cancer.^29^ Generally, major sources of LA include vegetable oils, seeds, nuts, meats, and eggs. However, it is the predominant PUFA in other foods with low fat content, such as low-fat dairy, vegetables, fruits, and grains.^30^ Therefore, the overall LA intake of individuals comes from different food sources. When the exposure is an individual nutrient, controlling other nutrients as confounders could be a reasonable approach to investigate the independent association. However, adjusting for food groups or overall diet quality might result in the over-adjustment of the relationship and conceal the association between exposure and outcome. Therefore, in the current analysis, we have considered some nutrients (MUFA, SFA, TFA and vitamin E), but not food groups or overall diet quality. This approach was also applied by previous studies investigating LA in relation to breast cancer.^22,23^

 The exact mechanisms by which LA intake might reduce the risk of breast cancer are unknown. Earlier studies have shown that chronic inflammation plays an important role in the development and progression of cancer, particularly breast cancer.^31^ Concerns about recommendations for LA-rich diets have been based on the assumption that arachidonic acid (AA) is generated through the metabolism of LA. AA is the main precursor of eicosanoids with inflammatory properties that might contribute in the pathogenesis of cancer,^32^ although anti-inflammatory eicosanoids derived from AA have also been found.^33^ Moreover, multiple studies indicate that variations in dietary LA have little effect on circulating arachidonic acid levels.^34,35^ A meta-analysis of interventional studies found that addition of LA to the diet did not increase inflammatory biomarkers, including C-reactive protein, cytokines, fibrinogen, soluble vascular adhesion molecules, plasminogen activator inhibitor type 1, or tumor necrosis factor-α among healthy participants.^36^ Indeed, earlier studies indicated that higher intakes of LA might improve insulin sensitivity, which can affect the risk of breast cancer. Dietary LA intake has also been associated with lower concentrations of inflammatory factors such as C-reactive protein, interleukin 6 (IL-6), and IL-1β. This can further explain the favorable association between dietary LA and breast cancer risk.^32^ More studies are needed to determine the exact role of LA in the etiology of breast cancer.

 The strengths of this study are the large number of participants and the ability to adjust for a wide range of potential confounders to obtain an independent association. We used energy-adjusted intakes of LA in the current study, which can help reduce misclassification of study participants. Furthermore, validated questionnaires were applied for data collection, which can further support the accuracy of the findings. However, some limitations need to be considered. First, the causality of the associations cannot be established from this study because of its case-control design. Second, although we controlled for measured lifestyle and prognostic factors, unknown or unmeasured confounders cannot be excluded due to the study’s observational nature. Third, we estimated dietary intake of LA using FFQ and measurement errors such as underreporting of dietary intakes are inevitable. Fourth, our findings are subject to selection and recall biases, which are common in case-control studies. Fifth, cases and controls were retrospectively interviewed about their LA intake. Bias could potentially be introduced if the cases altered their diet after diagnosis of cancer or in response to anti-cancer treatment. To reduce this bias, cases were asked to report their usual frequency of intake in the year before their diagnosis. We also excluded individuals with other medical disorders that might change dietary habits. Given that food intake varies among individuals, we cannot estimate how much possible changes in diet affect LA intake. Moreover, there is a long period between dietary exposure and the onset of breast cancer. Dietary changes prior to the 1-year mark would alter the association between LA intake and breast cancer risk. However, epidemiological studies have shown that adults generally maintain stable and long-term dietary habits.^37,38^ Cases and controls were matched in terms of age using age groups. According to this method, we equalized the number of cases and controls in each age group. However, there was a significant difference in the mean age between cases and controls. In such conditions, matching has not removed age confounding and matching factors need to be controlled in the analysis.^39^ Moreover, there were significant differences between cases and controls regarding potential confounders like smoking and alcohol consumption. To handle this problem, we controlled for such confounders in the analysis. Lastly, our findings might largely relate to the Middle Eastern population and less generalizability can be considered with regard to other populations with different characteristics and dietary intakes.

## Conclusion

 In conclusion, we found that a high intake of LA was associated with lower odds of breast cancer. Such a significant inverse association was also seen in normal-weight women but not in overweight or obese individuals. Moreover, dietary LA was associated with lower odds of breast cancer among premenopausal women but not postmenopausal women. To confirm these findings, additional studies should be conducted in diverse populations.

## Competing Interests

 The authors declare no conflict of interest.

## Ethical Approval

 This study was conducted according to the guidelines laid down in the Declaration of Helsinki and all procedures involving human subjects/patients were approved by the Bioethics Committee of Tehran University of Medical Sciences, Tehran, Iran (Ethics code: IR.TUMS.VCR.REC.1397.1036).

## Funding

 There was no funding for this study.
